# Fracture Behavior and Mechanisms of Wheat Kernels Under Mechanical Loading

**DOI:** 10.3390/foods14183174

**Published:** 2025-09-12

**Authors:** Yu Chen, Sen Ma, Xiaoxi Wang, Xiaoling Tian

**Affiliations:** 1 College of Food Science and Engineering, Henan University of Technology, Zhengzhou 450001, China; chenyu@haut.edu.cn (Y.C.); masen@haut.edu.cn (S.M.); wangxxly@163.com (X.W.); 2 College of Food Science and Technology, Henan Agricultural University, Zhengzhou 450002, China

**Keywords:** wheat kernel fracture mechanics, endosperm microstructure, bran layer mechanics, fractal dimension analysis

## Abstract

Wheat milling efficiency and flour quality are fundamentally governed by kernel fracture behavior during mechanical processing. This study systematically investigated the fracture characteristics of wheat kernels through a multi-stage experimental approach. Rupture tests comparing shear and compression loading revealed that shear reduced fracture energy by 40%, with vitreous kernels (16.13 mJ) showing greater resistance than floury types (10.45 mJ) at 13% moisture. Microstructural characterization revealed distinct fracture modes: vitreous kernels fractured intercellularly, while floury kernels fractured intracellularly—quantified via fractal geometry (vitreous: fractal dimension D = 1.262; floury: D = 1.365). Controlled bran removal experiments demonstrated that outer bran layers provide 40% of total fracture resistance, with vitreous kernels depending primarily on endosperm properties beyond 5% peeling, whereas floury kernels exhibited progressive strength loss with each layer removed. These findings enable optimized milling strategies: shear-based systems for energy efficiency, minimal processing (≤5% bran removal) for vitreous wheat, and moderate peeling (≤10%) for floury wheat, ultimately advancing both scientific understanding and industrial practice in cereal processing.

## 1. Introduction

The wheat milling process represents a critical mechanical operation in cereal processing, where the efficient separation of endosperm from bran layers determines both processing economics and product quality. This operation fundamentally exploits the differential mechanical properties between grain tissues, where bran layer adhesion governs separation efficiency and endosperm fracture resistance dictates energy requirements [1,2]. While hydrothermal conditioning can modulate these properties, current research has overemphasized bran-endosperm detachment while neglecting fracture-induced modifications to internal endosperm structure—a critical gap impacting milling yield and flour functionality. Recent advances in food material science suggest these internal structural changes may significantly influence starch damage and protein functionality in final flour products. Furthermore, fractal geometry has emerged as a powerful tool for quantifying complex fracture patterns in biological materials, yet its application to cereal grain fracture remains limited.

From a materials perspective, grain hardness serves as the primary quality parameter for milling performance, exhibiting strong correlation with endosperm microstructure [3,4]. Additionally, endosperm texture varies between vitreous and floury wheat, classified based on the compactness of endosperm tissue. Vitreous grains exhibit a translucent, glassy cross-section, whereas mealy grains display opaque, chalky endosperm [5,6]. These visual differences, attributed to cultivation, growth, and drying conditions rather than genetics, often correlate with protein content—vitreous wheat is typically high-protein and hard, while floury wheat tends to be low-protein and often exhibits a softer texture. Although vitreosity and hardness frequently coincide, they arise from distinct mechanisms: hardness is genetically controlled by puroindoline proteins, while vitreosity is influenced by environmental factors such as drying conditions and nitrogen availability [7,8,9].

Complementing the endosperm mechanical properties, wheat bran comprises multiple histologically distinct layers—the pericarp (including epidermis, hypodermis, and cross/tube cells), testa, nucellar layer, and aleurone layer [10,11,12]. These layers are defined by their embryonic origins and developmental roles [13,14]. Each layer contributes differently to the overall mechanical stability of the grain [15]. For instance, the outer pericarp provides rigidity, while the aleurone layer, though nutritionally valuable, can act as a fracture boundary during milling [16]. Previous studies have characterized the bulk mechanical properties of bran [17], but the role of individual layers in modulating grain breakage remains unclear. This knowledge gap hinders the optimization of milling processes, particularly for whole-grain or high-extraction flours where controlled bran fragmentation is essential [18,19,20].

This study investigates the fracture mechanisms of vitreous and floury wheat grains under varying stress modes, analyzing crack propagation within the endosperm and correlating findings with industrial milling outcomes. Furthermore, by systematically removing distinct bran layers and conducting controlled fracture tests, we elucidate the mechanical contributions of each layer to grain breakage. Our work aims to decode the fracture behavior of grains with divergent endosperm structures, assess the role of bran layers in stress distribution during breakage, and establish a tissue-level understanding of wheat grain fracture to optimize milling processes.

## 2. Material and Method

### 2.1. Material

The vitreous endosperm wheat grains (Zhengmai 366, harvested in Anyang, China) and online-flour materials in the milling systems were obtained from the milling workshop of a company in Zhengzhou, China. The floury endosperm wheat grains (Zhoumai 27, harvested in Zhoukou, China) and online-flour materials in the milling systems were obtained from the milling workshop of a company in Xinyang, China.

### 2.2. Sample Preparation

Wheat conditioning was performed by dividing vitreous wheat kernels and floury wheat kernels into three portions each and tempering at room temperature with target moisture of 13.0%, 14.5% and 16.0%.

Samples of wheat kernels with different degrees of peeling were prepared using a flexible wheat peeling and cleaning machine (Furongda-RCMTK, Henan Rongcheng Mechanical Engineering Co., Ltd., Zhengzhou, China) according to the method of Cai [21]. The tempering time and peeling duration were adjusted to achieve target peeling degrees of 0%, 5%, 10%, and 20% by mass. The peeled kernels were cleaned and stored in sealed containers for later use. The degree of peeling was calculated by the following formula:

(1) Degree of peeling (%) = (m_1_ − m_2_)/m_1_

where m_1_ (g) is the mass of wheat kernels before peeling and m_2_ (g) is the mass of wheat kernels after peeling.

### 2.3. Rupture Tests of Wheat Grains Under Different Deformation Modes

Rupture tests were carried out on grains of different moisture levels using a TMS-PRO physical analyzer, based on the method of Dobraszczyk et al. [2] with minor modifications. Wheat kernels were placed in the center of the load plate of the physical properties analyzer with the grooves facing downward (to minimize the interference of external forces), and the position of the kernels was adjusted so that their middle part was precisely aligned with the probe. Instrument parameters were set as follows: pre-test speed 1 mm/s, test speed 1 mm/s, post-test speed 1 mm/s, trigger force 0.75 N, distance 10 mm, and data acquisition rate 10 Hz. Shear rupture resistance was measured using a lightweight single-blade shear probe (tangential width 80 mm, blade diameter 0.3 mm), while compression resistance was determined using an aluminum cylindrical probe (diameter 21 mm). Thirty kernels were selected from each mode.

### 2.4. Scanning Electron Microscopy (SEM) Microstructure of Grain Fracture Surfaces and Milling System Materials

Samples with intact fracture surfaces (natural fracture surfaces after stress, not cut by the probe) of wheat kernels in the rupture experiments were selected. The break system products and starching mill feedstock from online-flour materials with different endosperm kernels were obtained. Samples were sputter-coated with gold (Quorum Q150R ES, Quorum Technologies Ltd., Laughton, UK) and observed using a scanning electron microscope (Quanta-250FEG, Thermo Fisher Scientific, Brno, Czech Republic ) at an accelerating voltage of 5 kV.

### 2.5. Confocal Laser Scanning Microscopy (CLSM) Microstructure of Different Peeling Degrees Wheat Grains

Wheat kernel sections with varying peeling degrees were prepared for microscopy following a modified protocol based on De-Brier et al. [22]. Samples were fixed in 1% glutaraldehyde for 48 h, dehydrated through an ethanol gradient (70%, 80%, 90%, 95% *v*/*v*, anhydrous ethanol), embedded in paraffin, and cryo-fixed at −20 °C for ≥24 h. Sections (20 μm thickness) were mounted on adhesive slides, stained sequentially with 1% *w*/*v* acid fuchsin and 0.01% *w*/*v* calcofluor white for 2 min, rinsed, air-dried. Sections were visualized using a confocal laser scanning microscope (Olympus V30000, Olympus Corporation, Tokyo, Japan) with excitation wavelengths of 488 nm and 405 nm for acid fuchsin and calcofluor white, respectively.

### 2.6. Breakage Tests on Wheat Grains with Different Peeling Degrees

Wheat kernels with varying peeling degrees were equilibrated to the same moisture content and subjected to breakage tests using a TMS-PRO analyzer fitted with a needle probe (Pre-test speed 1 mm/s, test speed 1 mm/s, post-test speed 1 mm/s, trigger force 3.80 N, distance 10 mm, and data acquisition rate 10 Hz). At least thirty replicates were performed per treatment.

### 2.7. Fractal Model

The fractal dimension D was calculated based on the model by Li [23] and Sun et al. [24], using Equations (2)–(4) as described in the results.

(2) *D =* ln*N*/ln(1/*r*)

(3) *N* = *L_i_*/*ε_i_*

(4) *r* = *ε_i_*/*L_oi_*

where *N* is number of generator segments, *r* is similarity ratio, *L_i_* is microscopic crack length, *ε_i_* is measurement scale and *L_oi_* is macroscopic reference.

### 2.8. Statistical Analysis

The rupture resistance force data were grouped into ten sets of three consecutive measurements, with the mean value of each group represented and then used for heatmaps were normalized to facilitate visual comparison. All other data obtained were subjected to analysis of variance (ANOVA) and evaluated by Duncan’s method. The significant difference was set at *p* < 0.05. Graphs were generated and analyzed using Origin Pro 8.5 (OriginLab Corp., Northampton, MA, USA).

## 3. Results and Discussion

### 3.1. Fracture Characteristics of Wheat Kernels with Different Endosperm Structures

**Figure 1:**
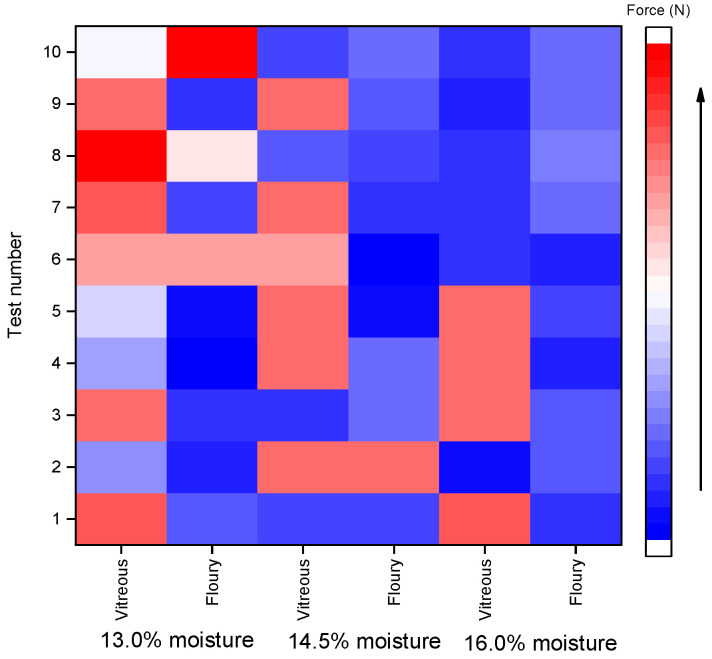
Heat map of shear rupture resistance force of wheat grains.

**Figure 2:**
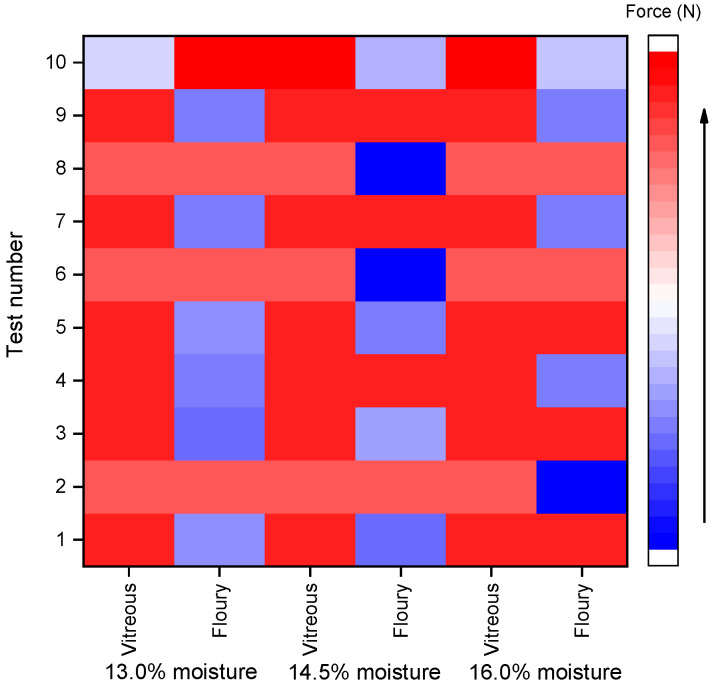
Heat map of compression rupture resistance force of wheat grains.

The heatmaps (Figure 1 and Figure 2) were generated based on normalized rupture resistance force data to visually compare the differences between shear and compression modes across various moisture levels (13.0%, 14.5%, and 16.0%) and kernel types (vitreous and floury). The color gradient from blue to red represents the magnitude of the rupture force, with blue indicating lower values and red indicating higher values. As illustrated in the heatmaps, compared with the shear mode, the compression mode shows more red-colored areas in the heatmap; in other words, the force required for resisting compressive rupture is generally higher across both kernel types. This indicates that the kernels are more susceptible to shear-induced fracture. Notably, shear resistance decreased with increasing moisture content, whereas compressive strength remained relatively stable between 14.5% and 16.0% moisture, which aligns with industry standards for hard wheat tempering (NY/T 1094.1-2006 [25]).

**Figure 3:**
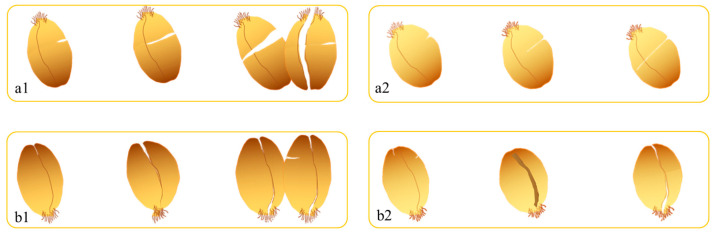
Breakage indication of wheat grain under mechanical force (**a1**) Vitreous endosperm kernels after shear rupture. (**a2**) Floury endosperm kernels after shear rupture. (**b1**) Vitreous endosperm kernels after extrusion rupture. (**b2**) Floury endosperm kernels after compression rupture. The moisture content of grains from left to right in each graph is 13.0%, 14.5%, and 16.0%, respectively).

The fracture patterns (Figure 3) revealed distinct failure modes. It shows that shear stress induced radial cracking (along the crease) with complete cleavage, particularly at 16.0% moisture. Compressive stress caused combined radial and axial cracking (perpendicular to the crease), with limited fragmentation at lower moisture levels (13.0–14.5%). These observations suggest that shear loading is more effective for complete kernel disintegration, while compression promotes controlled fracturing—a critical consideration for milling optimization.

Fracture energy analysis (Table 1) further highlighted structural differences. Vitreous kernels exhibited significantly higher shear (*p* < 0.05) and compressive fracture energy than floury kernels, attributable to their dense protein-starch matrix [9,26]. Shear energy decreased markedly with moisture (16.13 mJ at 13.0% and 10.21 mJ at 16.0%), whereas compressive energy showed no significant variation (~26 mJ). Floury kernels demonstrated lower shear energy (10.45–12.01 mJ) with no moisture-dependent trend (*p* < 0.05), but compressive energy declined slightly (25.03 mJ to 20.94 mJ), likely due to enhanced plasticity at higher hydration [2]. The inverse relationship between moisture content and shear resistance agrees with findings from maize kernel breakage studies [27,28], suggesting some commonality in water plasticization effects across cereal grains despite differences in kernel architecture and composition. However, the negligible moisture effect on vitreous kernels under compression implies that their fracture mechanics are governed more by protein-starch adhesion than by hydration—a finding corroborated by FEM simulations in similar cereal systems [29,30].

The lower energy requirement for shear-induced fracture (10–16 mJ and 21–26 mJ for compression) suggests that shear-based preprocessing (e.g., toothed roller milling) could enhance breakage efficiency, especially for vitreous wheat. However, the moisture insensitivity of floury kernels under shear necessitates alternative strategies, such as targeted tempering, to optimize fragmentation. These insights bridge material science and milling technology, offering a framework for kernel-specific process design.

### 3.2. Fractal Modeling of Stress Crack Propagation in Wheat Endosperm with Different Structures

#### 3.2.1. Microstructural Characterization of Fractured Kernels

**Figure 4:**
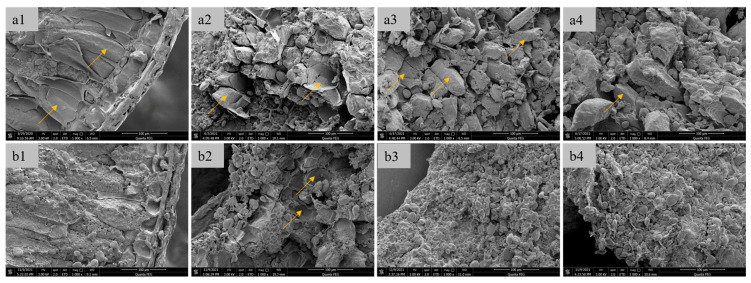
SEM images of wheat grain cross-section and online-flour materials ((**a1**, **a2**, **a3**, and **a4**), respectively, represent the cross-sectional, the materials ground by the 1B mill, the materials ground by the 3B mill and the feed materials to the starching mill of vitreous grain; While (**b1**, **b2**, **b3**, and **b4**), respectively, represent the cross-sectional, the materials ground by the 1B mill, the materials ground by the 3B mill and the feed materials to the starching mill of floury grain). The yellow arrows point to the cell wall of the endosperm.

Scanning electron microscopy (SEM) analysis of vitreous and floury wheat kernels revealed distinct endosperm fracture patterns (Figure 4). Vitreous endosperm exhibited a honeycomb-like cellular structure with intact cell walls (>85% integrity index, Figure 4(a1)), stabilized by arabinoxylan-protein matrices in the intercellular space. In contrast, floury endosperm displayed deconstructed tissue morphology, characterized by exposed starch granules (62.3 ± 5.1%) and fragmented cell walls (Figure 4(b1)), consistent with the weakened barrier effect reported by Korompokis et al. [31]. Intermediate milling products (e.g., break and starching system stocks) further highlighted these differences. Vitreous kernels retained prismatic endosperm cells (Figure 4(a2–a4)), while floury kernels yielded only cell-wall debris and free starch granules (Figure 4(b2–b4)). The observed behavior is consistent with the protein-starch adhesion theory, where endosperm cohesion is governed by covalent bonds, electrostatic forces, and hydrophobic interactions [32]. These molecular interactions, including van der Waals forces, ionic bonds, and molecular entanglement, similarly regulate the adhesion between major endosperm components. In wheat endosperm, cellular adhesion is mediated by cell walls containing approximately 15% protein and 75% non-starch polysaccharides (primarily cellulose, hemicellulose, and pentosans) [33]. The elevated protein content in vitreous endosperm strengthens intercellular bonding, resulting in preferential crack propagation along cell boundaries. In contrast, floury endosperm exhibits weaker intramolecular forces, leading to transcellular fracture and the characteristic step-like cleavage morphology.

#### 3.2.2. Fracture Mechanics and Crack Propagation

**Figure 5:**
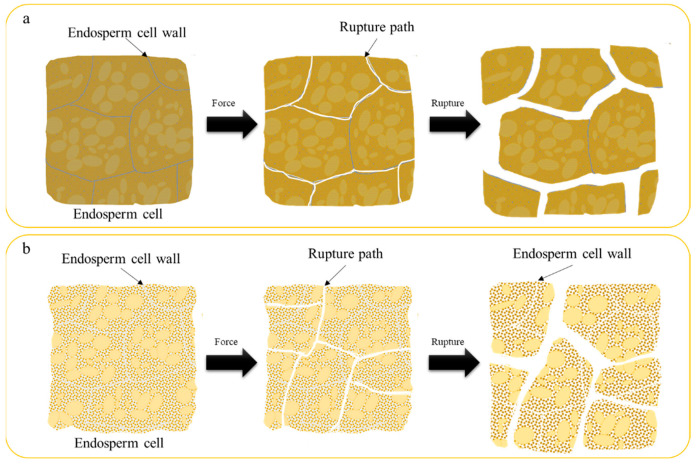
Diagram of wheat grain internal rupture ((**a**): Vitreous endosperm kernels. (**b**): Floury endosperm kernels).

Fracture paths diverged significantly between kernel types. Vitreous kernels fractured intercellularly (Figure 5a), following cell-wall interfaces due to weaker intercellular adhesion [1]. Floury kernels exhibited intracellular cracking (Figure 5b), with randomized paths reflecting inhomogeneous starch-protein bonding [32].

This behavior correlates with hardness and hydration effects. Stress concentration at crack tips preferentially exploits the weakest molecular linkages—intercellular hemicelluloses in vitreous wheat versus starch-protein interfaces in floury wheat [2]. Notably, floury kernels’ fragmented cell walls may enhance digestibility by reducing physical barriers to enzymatic access [31], suggesting applications in tailored food products.

Previous research has investigated wheat crushing through various mechanical loading methods including roller grinding, impact, and shear. Among these studies, Campbell and Webb [34] applied the crushing matrix method to wheat milling and extended the method to non-square matrices covering different sizes for input and output particle size distribution and proposed a first-order fracture equation. Campbell et al. [35,36] investigated the fracture equation and its continuous functional form, ultimately developing a refined secondary fracture equation. This enhanced model incorporated key factors influencing crushing efficiency, including wheat hardness, kernel morphology, grain damage, moisture content, and roller configuration, thereby improving both interpretability and generalizability. Molecular-level mechanisms may further explain the observed fracture behavior. The formation of bran dietary fiber-gluten complexes [37] and polysaccharide-mediated water regulation in protein networks [38] likely contributes to the enhanced mechanical integrity of vitreous kernels and their differential response to hydration. Even so, the research still needs a large number of wheat grain sample information to fill the database so that it does not only stay in the theoretical function stage. Based on the actual situation of wheat in China, the difference and influence of vitreous and floury grains should also be considered in the follow-up research.

#### 3.2.3. Fractal Model Development

The endosperm cells of wheat grains can be divided into marginal cells, prismatic cells and central cells according to shape and size: marginal cells are similar to aleurone layer cells in shape and size, with an average diameter of about 60 μm, distributed in one or two layers adjacent to the aleurone layer cells; prismatic cells are shaped like their names, with approximately rectangular cross-sections, about 120 μm in length and 40 μm in width, and are located on the inside of marginal cells. The central cells accounted for the largest proportion of the three kinds of cells, distributed from the inside of the prismatic cells to the center of the grain, with a polygonal cross-section, about 72–144 μm in length and 69–120 μm in width [39]. The length of wheat grain is about 5 mm and the width is 2 mm. The average thickness of cortex is about 100 μm.

**Figure 6:**
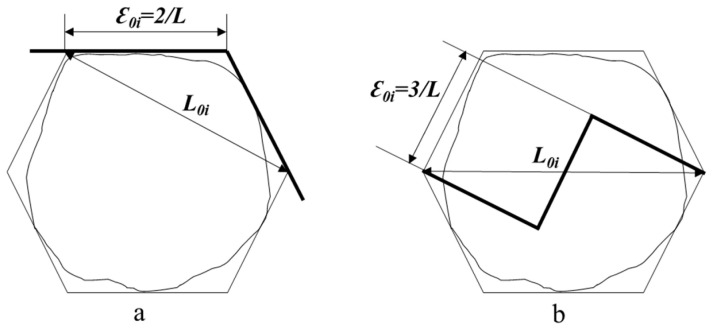
Stress crack propagation model of wheat grain ((**a**): Vitreous endosperm kernels. (**b**): Floury endosperm kernels).

As kernel fractures typically propagate into central cells ( Figure 2 ), their hexagonal cross-sections (inscribed circle diameter: 35–70 μm) provide a geometric basis for fractal analysis. To quantify the fractal complexity of fracture patterns, we applied the fractal model using Equations (2)–(4). Calculation for vitreous wheat kernels, the calculation process was as follows (Figure 6a):

(5) *D* = ln*N*/ln(1/*r*) = ln2/ln√3 ≈ 1.262

(6) *D* = ln*N*/ln(1/*r*) = ln3/ln√5 ≈ 1.365

Calculation the floury wheat kernels, the calculation process was as follows (Figure 6b):

The lower D for vitreous kernels indicates simpler, cell-boundary-localized cracks, while floury kernels’ higher D reflects chaotic intracellular propagation. These results align with Sutton [40] cleavage fracture theory, in which material heterogeneity governs crack propagation randomness. The model bridges microstructure and milling outcomes. Vitreous wheat benefits from shear-dominated milling (e.g., corrugated roller mills) to exploit intercellular weakness. Floury wheat requires compressive/impact forces to overcome its stochastic fracture behavior.

The fracture mechanisms characterized in this study may exhibit both parallels and divergences when compared to other cereal grains. Similarly to wheat, maize kernels also demonstrate moisture-dependent fracture behavior and endosperm texture variations (vitreous and floury) that influence their mechanical properties [28]. However, notable differences exist in kernel morphology and structural organization across cereal species. For instance, rice kernels possess a more rigid hull structure that significantly influences fracture initiation [41], while barley kernels exhibit distinct cell wall composition that may alter crack propagation patterns [42]. The fractal approach developed here for quantifying fracture complexity could potentially be adapted to other cereal systems, though specific model parameters would require adjustment to account for species-specific anatomical features [2]. Future comparative studies across multiple cereal species would help establish the generalizability of these fracture mechanisms and modeling approaches.

### 3.3. Bran Layer Contributions to Kernel Integrity

**Figure 7:**
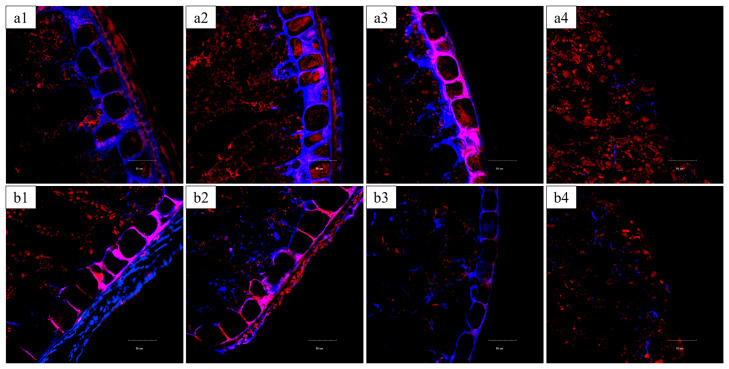
CLSM images of bran layers in wheat kernels with different degrees of peeling. ((**a1**–**a4**) Vitreous endosperm kernels at peeling degrees of 0%, 5.01%, 10.22%, and 18.46%, respectively; (**b1**–**b4**) Floury endosperm kernels at peeling degrees of 0%, 4.75%, 9.37%, and 19.33%, respectively. Polysaccharides in cell walls are stained blue-purple (Calcofluor White), and proteins are stained red (Acid Fuchsin)).

**Figure 8:**
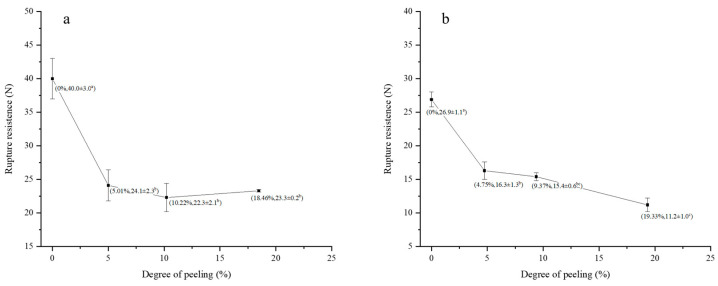
Breakage resistance of wheat kernels with different degrees of peeling. (**a**): Vitreous endosperm kernels. (**b**): Floury endosperm kernels. Different letters above bars indicate significant differences (*p* < 0.05) among peeling degrees for each kernel type.

Systematic peeling experiments, combined with CLSM, were conducted to precisely track the removal of specific bran layers (Figure 7) and evaluate their individual contributions to kernel mechanical integrity (Figure 8).

In vitreous kernels, the removal of the outer layers (5.01% mass loss, Figure 7(a2)) corresponded primarily to the loss of the outer pericarp layers (epidermis and hypodermis), which are characterized by thick, heavily blue-stained cell walls. This structural change coincided with a significant 40% reduction in fracture resistance (from 40.0 ± 3.0 N to 24.1 ± 2.3 N; *p* < 0.05), as quantified in Figure 8a, highlighting these outer layers’ role as the primary mechanical barrier, consistent with the findings of Antoine et al. [16]. Further peeling to 10.22% (Figure 7(a3)) removed the intermediate layers (cross cells, tube cells, and testa), evident by the diminished blue-stained area. However, no further significant decrease in rupture force occurred (22.3 ± 2.1 N; *p* ≥ 0.05), indicating that the mechanical properties beyond this point are governed predominantly by the underlying endosperm. Peeling to 18.46% (Figure 7(a4)), which included removal of the aleurone layer, did not significantly alter the mechanical strength (23.3 ± 0.2 N), supporting the notion that the aleurone contributes minimally to bulk mechanical properties [15,16].

A contrasting progressive failure mechanism was observed for floury kernels. The initial peel to 4.75% (Figure 7(b2)), which removed the outer pericarp, led to a similar 40% strength reduction (from 26.9 ± 1.1 N to 16.3 ± 1.3 N; Figure 8b). Subsequent peeling to 9.37% (Figure 7(b3)), which removed the intermediate layers, resulted in a further significant strength loss (to 15.4 ± 0.6 N; *p* < 0.05), suggesting all bran layers contribute to the structural integrity of floury kernels. Complete removal of 19.33% of the bran, including the aleurone layer (Figure 7(b4)), caused severe structural compromise, and evident a final significant reduction in strength to 11.2 ± 1.0 N, suggesting a greater integrated contribution of all bran layers to the structural integrity of floury kernels [31].

These results, correlating specific layer removal at precise peeling degrees with mechanical response, demonstrate that the outer pericarp provides the major mechanical contribution for both kernel types. However, the inherent structural characteristics of the floury endosperm make its integrity more dependent on the support of all bran layers, leading to a progressive strength decline with peeling.

The mechanical contribution of each bran layer, as quantified in this section, provides a tissue-level mechanistic explanation for the macroscopic fracture behaviors observed in Section 3.1. Combined with the earlier finding that shear loading is significantly more energy-efficient than compression, these results strongly support the strategic prioritization of shear-based break systems (toothed rollers) in milling operations. For vitreous wheat, the process should emphasize endosperm fragmentation within the break system, as bran removal beyond ~5% provides diminishing returns in energy savings, consistent with the minimal change in rupture resistance observed post-initial peeling. In contrast, for floury wheat, moderate peeling limited to ≤10% is recommended. This strategy achieves an optimal balance by leveraging the progressive reduction in kernel strength with layer removal to conserve energy, while simultaneously retaining the nutritionally valuable inner bran components.

## 4. Conclusions

This study systematically elucidates the fracture behavior of wheat kernels through an integrated multi-scale approach. Experimental results demonstrate that endosperm texture fundamentally determines fracture mechanisms, with vitreous kernels exhibiting superior mechanical integrity due to their continuous protein-starch matrix, whereas floury kernels display reduced fracture energy and more disordered crack propagation. Peeling experiments further quantify the mechanical contribution of the bran layers, revealing that the outer pericarp accounts for approximately 40% of the total kernel strength. Certain limitations should be acknowledged. The single-kernel testing method, although enabling precise tissue-level analysis, does not fully represent industrial milling conditions. Furthermore, only two wheat varieties were examined in this study, and future investigations should incorporate a broader range of genetic diversity. Subsequent research should prioritize pilot-scale validation of these findings, particularly focusing on the interactions between kernel-type-specific processing parameters and overall system performance within industrial milling environments.

The analytical approaches developed in this study provide new insights into wheat kernel fracture mechanisms. The application of fractal model offers a quantitative method for characterizing fracture patterns, while the mechanical quantification of individual bran layers contributes to understanding their functional roles. These findings suggest potential applications in optimizing milling processes based on kernel structural characteristics, particularly regarding energy efficiency considerations.

## Figures and Tables

**Table 1:** Fracture energy of wheat grains.

Type of Kernels	Moisture Content (%)	Shear Resistance Fracture Energy (mJ)	Compression Resistance Fracture Energy (mJ)
Vitreous	13.0	16.13 ± 2.91 ^a^	26.41 ± 5.24 ^a^
	14.5	14.11 ± 2.14 ^b^	26.36 ± 4.28 ^a^
	16.0	10.21 ± 1.61 ^c^	25.37 ± 4.98 ^a^
Floury	13.0	10.45 ± 1.74 ^a^	25.03 ± 4.47 ^a^
	14.5	10.83 ± 1.26 ^a^	23.71 ± 3.83 ^ab^
	16.0	12.01 ± 1.33 ^a^	20.94 ± 4.33 ^b^

Note: Different lowercase letters in the data of the same column for the same type of kernels indicate significant differences (*p* < 0.05).

## Data Availability

The original contributions presented in the study are included in the article/Supplementary Materials, further inquiries can be directed to the corresponding author.

## Supplementary Materials

The following supporting information can be downloaded at https://www.mdpi.com/article/10.3390/foods14183174/s1: Table S1: Proximate composition of the vitreous and floury wheat kernels (dry basis, %).

## References

[1] Campbell G.M., Fang C., Muhamad I.I. (2007). On predicting roller milling performance VI: Effect of kernel hardness and shape on the particle size distribution from first break milling of wheat. Food Bioprod. Process..

[2] Dobraszczyk B.J., Whitworth M.B., Vincent J.F.V., Khan A.A. (2002). Single kernel wheat hardness and fracture properties in relation to density and the modelling of fracture in wheat endosperm. J. Cereal Sci..

[3] Gaines C.S., Raeker M.Ö., Tilley M., Finney P.L., Wilson J.D., Bechtel D.B., Martin R.J., Seib P.A., Lookhart G.L., Donelson T. (2000). Associations of starch gel hardness, granule size, waxy allelic expression, thermal pasting, milling quality, and kernel texture of 12 soft wheat cultivars. Cereal Chem..

[4] Gazza L., Taddei F., Corbellini M., Cacciatori P., Pogna N.E. (2008). Genetic and environmental factors affecting grain texture in common wheat. J. Cereal Sci..

[5] Evers A.D., Bechtel D.B., Pomeranz Y. (1988). Microscopic Structure of the Wheat Grain. Wheat Chemistry and Technology.

[6] Turnbull K.-M., Rahman S. (2002). Endosperm texture in wheat. J. Cereal Sci..

[7] Morris C.F. (2002). Puroindolines: The molecular genetic basis of wheat grain hardness. Plant Mol Biol..

[8] Gupta R.B., Masci S., Lafiandra D., Bariana H.S., MacRitchie F. (1996). Accumulation of protein subunits and their polymers in developing grains of hexaploid wheats. J. Exp. Bot..

[9] Pomeranz Y., Williams P.C., Pomeranz Y. (1990). Wheat Hardness: Its Genetic, Structural, and Biochemical Background, Measurement, and Significance. Advances in Cereal Science and Technology.

[10] Anderssen R.S., Haraszi R. (2009). Characterizing and exploiting the rheology of wheat hardness. Eur. Food Res. Technol..

[11] Delcour J.A., Hoseney R.C. (2010). Yeast-Leavened Products. Principles of Cereal Science and Technology.

[12] Wang H., Sun S., Ge W., Zhao L., Hou B., Wang K., Lyu Z., Chen L., Xu S., Guo J. (2020). Horizontal gene transfer of *Fhb7* from fungus underlies *Fusarium* head blight resistance in wheat. Science.

[13] Barron C., Parker M.L., Mills E.N.C., Rouau X., Wilson R.H. (2005). FTIR Imaging of wheat endosperm cell walls in situ reveals compositional and architectural heterogeneity related to grain hardness. Planta.

[14] Bechtel D.B., Abecassis J., Shewry P.R., Evers A.D., Khan K., Shewry P.R. (2009). Development, Structure, and Mechanical Properties of the Wheat Grain. Wheat: Chemistry and Technology.

[15] Peyron S., Chaurand M., Rouau X., Abecassis J. (2002). Relationship between bran mechanical properties and milling behaviour of durum wheat (*Triticum durum* Desf.). Influence of tissue thickness and cell wall structure. J. Cereal Sci..

[16] Antoine C., Peyron S., Mabille F., Lapierre C., Bouchet B., Abecassis J., Rouau X. (2003). Individual contribution of grain outer layers and their cell wall structure to the mechanical properties of wheat bran. J. Agric. Food Chem..

[17] Greffeuille V., Mabille F., Rousset M., Oury F.X., Abecassis J., Lullien-Pellerin V. (2007). Mechanical properties of outer layers from near-isogenic lines of common wheat differing in hardness. J. Cereal Sci..

[18] Hemery Y., Rouau X., Dragan C., Bilici M., Beleca R., Dascalescu L. (2009). Electrostatic properties of wheat bran and its constitutive layers: Influence of particle size, composition, and moisture content. J. Food Eng..

[19] Hermans W., Silventoinen-Veijalainen P., De Bondt Y., Langenaeken N.A., Nordlund E., Courtin C.M. (2024). Isolating a fraction enriched in sub-aleurone gluten proteins through dry fractionation of wheat miller’s bran. Innov. Food Sci. Emerg. Technol..

[20] Chen Z., Mense A.L., Brewer L.R., Shi Y.C. (2024). Wheat bran layers: Composition, structure, fractionation, and potential uses in foods. Crit. Rev. Food Sci. Nutr..

[21] Cai W.Y., Cai W.Y. (2021). Effects of Flexible Debranning on Wheat Grain and Flour Quality. Master’s Thesis.

[22] De Brier N., Gomand S.V., Donner E., Paterson D., Delcour J.A., Lombi E., Smolders E. (2015). Distribution of minerals in wheat grains (*Triticum aestivum* L.) and in roller milling fractions affected by pearling. J. Agric. Food Chem..

[23] Li D. (2001). Experimental Study and Mechanism Analysis of Stress Crack Formation, Propagation and Inhibition in Rice Drying. Ph.D. Thesis.

[24] Sun J.X., Guo Y.M., Cui Q.L., Xu B.H. (2020). Mechanism of crack initiation and propagation in millet seeds based on SEM and fractal theory analysis. J. Shanxi Agric. Univ..

[25] (2016). Wheat Experimental Milling Part 1: Equipment, Sample Preparation, and Tempering.

[26] Samson M.-F., Mabille F., Chéret R., Abécassis J., Morel M.-H. (2005). Mechanical and physicochemical characterization of vitreous and mealy durum wheat endosperm. Cereal Chem..

[27] Zhou Y., Dhital S., Zhao C., Ye F., Chen J., Zhao G. (2021). Dietary Fiber-Gluten Protein Interaction in wheat flour dough: Analysis, consequences and proposed mechanisms. Food Hydrocoll..

[28] Zhang H., Xu G. (2019). Physicochemical properties of vitreous and floury endosperm flours in maize. Food Sci. Nutr..

[29] Jia F., Wang J., Fan P., Yin H., Guan J., Zhou M. (2014). Analysis of finite element method on mechanical properties of wheat kernel. Interdiscip. Sci. Comput. Life Sci..

[30] Ren G.Y., Zhang S., Li L.L., Liu W.C., Cao W.W., Wei X.Y., Wu X.T., Xu D. (2025). Finite element analysis of wheat grain mechanical properties based on micro-CT modeling. Trans. Chin. Soc. Agric. Eng..

[31] Korompokis K., Brier N.D., Delcour J.A. (2019). Differences in endosperm cell wall integrity in wheat (*Triticum aestivum* L.) milling fractions impact on the way starch responds to gelatinization and pasting treatments and its subsequent enzymatic in vitro digestibility. Food Funct..

[32] Chichti E., George M., Delenne J.-Y., Lullien-Pellerin V. (2015). Changes in the starch-protein interface depending on common wheat grain hardness revealed using atomic force microscopy. Plant Sci..

[33] Merali Z., Collins S.R.A., Elliston A., Wilson D.R., Käsper A., Waldron K.W. (2015). Characterization of cell wall components of wheat bran following hydrothermal pretreatment and fractionation. Biotechnol. Biofuels.

[34] Campbell G.M., Bunn P.J., Webb C., Hook S.C.W. (2001). On Predicting roller milling performance: Part II. The breakage function. Powder Technol..

[35] Campbell G.M., Salman A.D., Ghadiri M., Hounslow M.J. (2007). Roller Milling of Wheat. Handbook of Powder Technology: Particle Breakage.

[36] Campbell G.M., Sharp C., Wall K., Mateos-Salvador F., Gubatz S., Huttly A., Shewry P. (2012). Modelling wheat breakage during roller milling using the double normalised kumaraswamy breakage function: Effects of kernel shape and hardness. J. Cereal Sci..

[37] Li M., Ma S., Zheng X., Li L. (2025). Studies on the formation mechanism and multiscale structure of wheat bran dietary fiber-gluten protein complex. Food Biosci..

[38] Li M., Ma S. (2025). From water-ice regulation to polysaccharides-protein assembly: Molecular mechanism of polysaccharides to improve the cryostability of gluten proteins. Food Res. Int..

[39] Matzke K., Riederer M. (1990). The composition of the cutin of the caryopses and leaves of *Triticum aestivum* L.. Planta.

[40] Sutton A.P. (2016). Interfaces in Crystalline Materials.

[41] Liu X., Shi Z., Zhang Y., Li H., Pei H., Yang H. (2024). Characteristics of damage to brown rice kernels under single and continuous mechanical compression conditions. Foods.

[42] Mohamed A.H., Omar A.A., Attya A.M., Elashtokhy M.M.A., Zayed E.M., Rizk R.M. (2021). Morphological and molecular characterization of some Egyptian six-rowed barley (*Hordeum vulgare* L.). Plants.

