# Supplementary material for: Fracture Behavior and Mechanisms of Wheat Kernels Under Mechanical Loading
Source: Foods. 2025 Sep 12;14(18):3174. doi: 10.3390/foods14183174 (PMC12468816; doi:10.3390/foods14183174)
Supplement: Supplementary file 1 [file foods-14-03174-s001.zip › foods-3825041-supplementary.pdf]

Table S1. Proximate composition of the vitreous and floury wheat kernels (dry basis, %).

Component	Vitreous kernels	Floury Kernels	Analytical Standard
Starch (%)	70.04±1.27^{b}	73.77±1.09^{a}	AACC 76-13.01
Crude protein (%)	13.93±0.03^{a}	10.91±0.01^{b}	AACC 46-13.01
Ash (%)	1.73±0.08^{a}	1.59±0.07^{a}	AACC 08-01.01

Note: Different lowercase letters in the data of the same row indicate significant differences (p<0.05).
